# Supplementary material for: Natural Variation in OASC Gene for Mitochondrial O-Acetylserine Thiollyase Affects Sulfate Levels in Arabidopsis
Source: Plants (Basel). 2022 Dec 21;12(1):35. doi: 10.3390/plants12010035 (PMC9824738; doi:10.3390/plants12010035)
Supplement: Supplementary file 1 [file plants-12-00035-s001.zip › plants-2123055-supplementary.pdf]

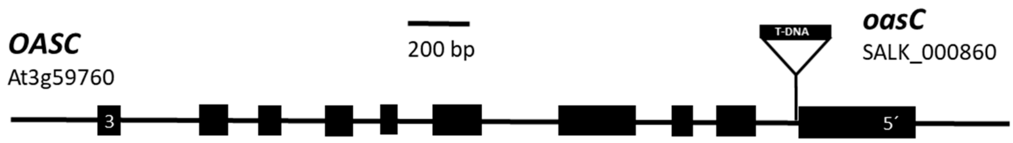

Supplementary Figure S1. Scheme of *oasC* T-DNA line.

```
OASC_B.napus     -MAMLMASRFNSEAKIASRFLSTLLRNQRVTASSSSMASASSSSALLLNPLTS----ST
OASC_A.thaliana  MVAMIMASRFNREAKLASQILSTLLGNRSCYTS-----MAATSSSALLLNPLTSSSSSST
                   ** ****** *** **  ***** *     *       * ************    **

OASC_B.napus     LRHFRSSPEISSLSFSAS-GFPLGMRSQQSRSYGDVSKRDP---CEAVKLETGADGLNIA
OASC_A.thaliana  LRRFRCSPEISSLSFSSASDFSLAMR-RQSRSFADGSERDPSVVCEAVKRETGPDGLNIA
                 ** ** **********    * * **  ****  * * ***   ***** *** ******

OASC_B.napus     QNVSQLIGKTPMVYLNSMAKGCVANIAAKLEIMEPCCSVKDRIGYSMVTDAEQKGFISPG
OASC_A.thaliana  DNVSQLIGKTPMVYLNSIAKGCVANIAAKLEIMEPCCSVKDRIGYSMVTDAEQKGFISPG
                  **************** ******************************************

OASC_B.napus     KSVLVEPTSGNTGIGLAFIAASRGYRLILTMPSSMSMERRVLLKAFGAELVLTDPAKGMT
OASC_A.thaliana  KSVLVEPTSGNTGIGLAFIAASRGYRLILTMPASMSMERRVLLKAFGAELVLTDPAKGMT
                 ******************************** ***************************

OASC_B.napus     GAVQKAEEILKSTPDAYMLQQFDNPANPKIHYETTGPEIWEDTKGKVDIFVAGIGTGGTI
OASC_A.thaliana  GAVQKAEEILKNTPDAYMLQQFDNPANPKIHYETTGPEIWDDTKGKVDIFVAGIGTGGTI
                 *********** **************************** *******************

OASC_B.napus     TGVGRFIKEQNPKVQVIGVEPTESDILSGGKPGPHKIQGIGAGFIPKNLDRAIMDEVIAI
OASC_A.thaliana  TGVGRFIKEKNPKTQVIGVEPTESDILSGGKPGPHKIQGIGAGFIPKNLDQKIMDEVIAI
                 ********* *** ************************************  ********

OASC_B.napus     SSEEAIETAKQLALKEGLMVGISSGAAAAAAIMVAKRPENAGKLIAVVFPSFGERYLSTP
OASC_A.thaliana  SSEEAIETAKQLALKEGLMVGISSGAAAAAAIKVAKRPENAGKLIAVVFPSFGERYLSTP
                 ******************************** ***************************

OASC_B.napus     LFQSIREEVEKMQPEI
OASC_A.thaliana  LFQSIREEVEKMQPEV
                 ***************
```

Supplementary Figure S2. Protein sequence comparison of *OASC* gene from *B. napus* and *A. thaliana*. Fully conserved residues are indicated in blue asterisks. The amino acid changes related to low sulfate accumulation are marked in red.

Supplementary Table S1. OASC haplotypes and sulfate content in *B. napus* varieties.

Haplotypes	SNP markers						Sulfate (µmol/g FW)
	JCVI_ 8073:282	JCVI_ 8073:288	JCVI_ 8073:354	JCVI_ 8073:377	JCVI_ 8073:588	JCVI_ 8073:649	
D75_INCxCON	-	-	R	R	S	C	22.6
D79_JETN	-	-	R	R	S	C	36.9
A42_Dar	-	-	R	R	S	C	27.8
A136_Roc	-	-	R	R	S	C	25.2
IB28_Cas	-	-	R	R	S	C	24.5
IB37_ColxNic	-	-	R	R	S	C	31.7
IB70_HanxGas	-	-	R	R	S	C	39.1
IB100_Maj	-	-	R	R	S	C	23.9
IB106_MoaR	-	-	R	R	S	C	23.6
TAP	K	C	R	R	-	C	35.6
D128_Q10	K	C	R	R	G	M	30.2
D6_APE	K	C	R	R	S	C	31.6
D7_APExGIN	K	C	R	R	S	C	23.5
D14_Bol	K	C	R	R	S	C	24.8
D26_CAPxMOH	K	C	R	R	S	C	22.1
D74_HURxNAV	K	C	R	R	S	C	18.8
D83_KAR	K	C	R	R	S	C	37.9
D102_MAT	K	C	R	R	S	C	21.9
A11_Bie	K	C	R	R	S	C	36.1
IB23_Can	K	C	R	R	S	C	32.2
IB29_Cat	K	C	R	R	S	C	14.2
IB50_DwaE	K	C	R	R	S	C	38.7
IB73_Hug	K	C	R	R	S	C	34.0
IB99_MadxRec	K	C	R	R	S	C	23.4
IB121_Palu	K	C	R	R	S	C	24.6
IB125_POH285B	K	C	R	R	S	C	15.8
IB139_Sam	K	C	R	R	S	C	27.1
IB151_SloK	K	C	R	R	S	C	39.8
D127_PRI	K	C	R	R	S	C	20.7
D131_Raf	K	C	R	R	S	C	29.6
D132_RAGJ	K	C	R	R	S	C	33.7
D137_ROCxLIZ	K	C	R	R	S	C	25.7
IB133_Ram	K	M	R	R	S	M	20.7
D146_SIBB	T	-	A	G	G	M	19.3
A144_She	T	-	R	R	S	M	38.7
IB21_Cab	T	C	R	R	S	C	34.0
IB52_EngG	T	C	R	R	S	C	25.4
D113_NEWH	T	M	-	G	G	M	-
IB86_LemM	T	M	A	G	G	-	30.0
IB111_N02D_1952	T	M	A	G	G	-	27.7
D3_ALT	T	M	A	G	G	M	21.3

D4_AMBxCOM	T	M	A	G	G	M	18.4
D10_BAL	T	M	A	G	G	M	16.5
D31_CES	T	M	A	G	G	M	17.4
D39_COR	T	M	A	G	G	M	32.6
D46_DIP	T	M	A	G	G	M	20.3
D55_EUR	T	M	A	G	G	M	13.6
D69_HAN	T	M	A	G	G	M	22.1
D108_MON	T	M	A	G	G	M	21.9
D170_TIN	T	M	A	G	G	M	28.7
D173_TOP	T	M	A	G	G	M	18.9
D181_WESD	T	M	A	G	G	M	20.1
A18_Bro	T	M	A	G	G	M	13.5
A25_Cap	T	M	A	G	G	M	25.9
A34_Chu	T	M	A	G	G	M	24.1
NIN	T	M	A	G	G	M	15.7
A155_Ste	T	M	A	G	G	M	21.8
A168_TEM	T	M	A	G	G	M	30.4
A176_Ver	T	M	A	G	G	M	32.6
A187_Xia	T	M	A	G	G	M	14.9
A188_Zho	T	M	A	G	G	M	24.8
IB02_AbuN	T	M	A	G	G	M	19.5
IB24_CanxCou	T	M	A	G	G	M	22.2
IB45_Dim	T	M	A	G	G	M	14.8
IB49_Dup	T	M	A	G	G	M	17.0
IB53_Erg	T	M	A	G	G	M	12.5
IB58_Exp	T	M	A	G	G	M	23.2
IB60_Fla	T	M	A	G	G	M	25.4
IB91_LicxExp	T	M	A	G	G	M	25.7
IB110_N01D_1330	T	M	A	G	G	M	23.3
IB120_Palm	T	M	A	G	G	M	26.1
IB130_Qui	T	M	A	G	G	M	28.3
IB141_SenNZ	T	M	A	G	G	M	25.1
IB143_ShaxWin	T	M	A	G	G	M	24.1
IB178_Vig	T	M	A	G	G	M	17.4
IB180_Vis	T	M	A	G	G	M	20.1
IB184_Yor	T	M	A	G	G	M	20.5
D117_NOR	T	M	A	G	G	M	29.2
D134_RAPCR	T	M	A	G	G	M	18.3
D182_WILR	T	M	A	G	G	M	20.9
IB164_Tai	T	M	A	G	G	M	24.1
A135_Reg	T	M	R	G	G	M	21.5
IB16_Bra	T	M	R	R	S	M	17.8
IB57_Exc	T	M	R	R	S	M	16.0

Supplementary Table S2. SNP markers in *OASC* gene in *B. napus*

SNP	P-value	Mutation
JCVI_8073:282	8.12×10^{-4}	Synonymous
JCVI_8073:288	3.28×10^{-5}	Synonymous
JCVI_8073:354	7.99×10^{-5}	Synonymous
JCVI_8073:377	4.87×10^{-5}	Non-synonymous
JCVI_8073:588	1.95×10^{-4}	Synonymous
JCVI_8073:649	2.05×10^{-5}	Synonymous

Supplementary Table S3. Primers used for qPCR and cloning

qPCR	Gene	Locus	Forward	Reverse
	UBC	AT5G25760	ctgcgactcagggaatcttc	ttgtgccattgaattgaacc
	APR1	At4G04610	cgatcaagtatccgtctgagaa	ggacaagattcaagaacgaagt
	APR2	At1G62180	aaaagagctccacgggctat	cgacatgagtgaatcaacatctc
	APR3	AT4G21990	ccaatcaagtatccatcagagaag	ccgaacaagattcaagaaagatg
	SDI1	AT5G48850	tccctgtggagacactcctt	ccatctccgggttcttctct
	GGCT2;1	AT5G26220	tccaccggagctatttgc	cgttccaagtactccattgct
	OAS-TL A	At4G14880	gaacagaacgcaaacgtcaa	tcttgtgaggacctggcttc
	OAS-TL B	At2G43750	tttggatctggctattgtagatga	tccagaagatataccaaccaacaa
	OAS-TL C	At3G59760	tgctggaaaactcatagctgtt	acctcaggctgcattttctc
	OAS-TL C1	At3G61440	aatctaaaaaccccaatgtcaaga	tgccaccgttgagtatgttg
cloning	OAS-TL C	At3G59760	CACCcgacgtgttggatttcagga	ttaaccgctgactctctca
mutagenesis	OAS-TL C	At3G59760	ctagcgatgaGgaggcagag	ctctgcctcCtcatcgctag
